# Ginsenoside Rg1 ameliorates diabetic cardiomyopathy by inhibiting endoplasmic reticulum stress‐induced apoptosis in a streptozotocin‐induced diabetes rat model

**DOI:** 10.1111/jcmm.12739

**Published:** 2016-02-12

**Authors:** Haitao Yu, Juan Zhen, Yang Yang, Jinning Gu, Suisheng Wu, Quan Liu

**Affiliations:** ^1^ Cardiology The First Hospital of Jilin University Changchun Jilin China

**Keywords:** ginsenoside Rg1, diabetic cardiomyopathy, endoplasmic reticulum stress, apoptosis

## Abstract

Ginsenoside Rg1 has been demonstrated to have cardiovascular protective effects. However, whether the cardioprotective effects of ginsenoside Rg1 are mediated by endoplasmic reticulum (ER) stress‐induced apoptosis remain unclear. In this study, among 80 male Wistar rats, 15 rats were randomly selected as controls; the remaining 65 rats received a diet rich in fat and sugar content for 4 weeks, followed by intraperitoneal injection of streptozotocin (STZ, 40 mg/kg) to establish a diabetes model. Seven days after STZ injection, 10 rats were randomly selected as diabetic model (DM) controls, 45 eligible diabetic rats were randomized to three treatment groups and administered ginsenoside Rg1 in a dosage of 10, 15 or 20 mg/kg/day, respectively. After 12 weeks of treatment, rats were killed and serum samples obtained to determine cardiac troponin (cTn)‐I. Myocardial tissues were harvested for morphological analysis to detect myocardial cell apoptosis, and to analyse protein expression of glucose‐regulated protein 78 (GRP78), C/EBP homologous protein (CHOP), and Caspase‐12. Treatment with ginsenoside Rg1 (10–20 mg/kg) significantly reduced serum cTnI levels compared with DM control group (all *P* < 0.01). Ginsenoside Rg1 (15 and 20 mg/kg) significantly reduced the percentage of apoptotic myocardial cells and improved the parameters of cardiac function. Haematoxylin and eosin and Masson staining indicated that ginsenoside Rg1 could attenuate myocardial lesions and myocardial collagen volume fraction. Additionally, ginsenoside Rg1 significantly reduced GRP78, CHOP, and cleaved Caspase‐12 protein expression in a dose‐dependent manner. These findings suggest that ginsenoside Rg1 appeared to ameliorate diabetic cardiomyopathy by inhibiting ER stress‐induced apoptosis in diabetic rats.

## Introduction

With the increasing prevalence of diabetes, diabetic complications have attracted considerable attention. Diabetic cardiomyopathy is defined as myocardial structural and functional abnormalities that are independent of hypertension, coronary heart disease, valvular heart disease and related cardiac disorders 1. The disease manifests itself as focal myocardial necrosis, caused by metabolic disturbances and microvascular complications, subclinical cardiac dysfunction and ultimately progresses to heart failure, arrhythmia, cardiogenic shock and sudden death. Diabetic cardiomyopathy is a major contributor to morbidity and mortality in diabetic patients 2. A recent epidemiological study reported a 1.1% prevalence of diabetic cardiomyopathy in the general population and 16.9% among diabetic patients. Further, the study revealed a 31% incidence rate of death or heart failure in patients with diabetic cardiomyopathy 3. Therefore, early detection and management of diabetic cardiomyopathy is a key priority in diabetes patients.

Diabetic cardiomyopathy is characterized by myocardial dilatation or hypertrophy, and impaired systolic or diastolic function of the left ventricle. The underlying mechanisms for the pathogenesis of diabetic cardiomyopathy are not completely understood. Diverse molecular mechanisms such as oxidative stress, inflammation, myocardial fibrosis, endoplasmic reticulum (ER) stress and apoptotic cell death have all been proposed as potential contributed factors in the pathogenesis of diabetic cardiomyopathy 4. Cardiocyte apoptosis has been demonstrated in the hearts of diabetic individuals 5 and in streptozotocin (STZ)‐induced diabetic rats 6, 7. Earlier studies have demonstrated ultrastructural evidence of swelling and dilation of ER in the diabetic myocardium model 8, 9. Endoplasmic reticulum stress leads to unfolded protein response that is considered to be an adaptive response aimed at attaining ER homeostasis. Further, ER stress‐induced apoptosis is known to play a key role in the pathogenesis and progression of diabetic cardiomyopathy 10, 11. Glucose regulated protein (GRP) 78 is considered as an early regulator of ER stress 12, 13. On persistence of ER stress, apoptotic processes are promoted by C/EBP homologous protein (CHOP) and Caspase‐12, and Jun pathway 14, 15, 16. Moreover, treatment with exendin‐4 17, metallothionein 18, irbesartan 19 and timolol 20 ameliorated diabetic myocardial lesions by reducing ER stress‐induced apoptosis. These findings suggest that ER stress may be a therapeutic target in diabetic cardiomyopathy.

Currently, there are no specific drugs for the treatment of diabetic cardiomyopathy. Panax ginseng has been used for the treatment of various diseases for thousands of years. Ginsenoside Rg1 is one of the most important ingredients in Panax ginseng 21. Cardiovascular protective effects of ginsenoside Rg1 have been attributed to its inhibition of cell apoptosis and reduction in oxidative stress 22. Studies investigating myocardial protective effects of ginsenoside Rg1 have mainly concentrated on hypertrophic cardiomyopathy 23, acute myocardial infarction 24, myocardial ischaemia/reperfusion 25. No previous study has investigated the effect of ginsenoside Rg1 on diabetic myocardial damages. The objective of this study was to investigate the effects of ginsenoside Rg1 on diabetic cardiomyopathy in STZ‐induced diabetic rats, and to investigate whether the cardioprotective effects of ginsenoside Rg1 were mediated through inhibition of ER stress‐induced apoptosis.

## Materials and methods

### Materials

The study protocol was approved by the Animal Care and Use Committee of the University of Jilin. Eighty male 4‐week‐old Wistar rats (200 ± 20 g) were purchased from the Animal Center of Jilin University (Animal Certificate number: SCXK‐Ji 2008‐0005). The rats were housed at a temperature of 23 ± 2°C and 55 ± 2% humidity. The animals had free access to tap water and normal rat diet prior to the start of the experiment. Ginsenoside Rg1 with high‐performance liquid chromatographic analysis of the purity of >98% was obtained from the College of Pharmacy of Jilin University. Streptozotocin was obtained from Sigma Chemical Co., St. Louis, MO, USA. terminal deoxynucleotidyl transferase dUTP nick end labelling (TUNEL) staining kit was obtained from Promega Co., Madison, WI, USA. Goat polyclonal anti‐GRP78, anti‐CHOP and anti‐Caspase‐12 were obtained from Abcam Biological Products Co. Ltd, Cambridge, UK.

### Experimental protocol

All rats were acclimatized to the environment for a period of 1 week before the start of the experiment. Out of the 80 Wistar rats, 15 rats were randomly selected as normal controls; another 65 rats received a high‐fat and sugar diet (base material 66.6%, sucrose 20%, 10% lard, 0.4% cholesterol, 3% egg yolk powder) for 4 weeks, followed by intraperitoneal injection of STZ 40 mg/kg in 0.1% pH 4.5 citric acid buffer solution to induce a diabetic model (DM). Blood glucose levels were determined with SureStepPlus Blood Glucose Meter (Johnson & Johnson Co., New Brunswick, NJ, USA). A successful induction of a DM was considered when, 7 days after STZ injection, the rat had blood glucose levels >16.7 mmol/l for three consecutive days. Eight rats died from complications of diabetes after STZ injection; two rats were ruled out for unsuccessful diabetic induction. The other 55 rats that developed diabetes were assigned to one of the following four groups using a random number table: DM (*N* = 10), ginsenoside Rg1 (10 mg/kg/day; *N* = 15), ginsenoside Rg1 (15 mg/kg/day; *N* = 15) and ginsenoside Rg1 (20 mg/kg/day; *N* = 15). Ginsenoside Rg1 was administered daily by intraperitoneal injection for 12 weeks. Rats in the normal control group received the same volumes of 0.9% saline solution.

### Sample collection and cardiac function measurement

After 12 weeks of treatment, all rats were killed under anaesthesia attained with intraperitoneal injection of 2 ml of 3% pentobarbital sodium. Images were obtained with the transducer placed on the rat's shaved chest in a left lateral decubitus position. Echocardiography was performed using Philips7500 with a 15‐MHz transducer (Sonos 7500, Amsterdam, NL). The left ventricular end diastolic diameter (LVEDD), left ventricular posterior wall thickness (LVPWD), left ventricular shortening fraction (LVFS) and left ventricular ejection fraction (LVEF) were measured by M type ultrasound. A pulsed Doppler method was applied to determine the E and A peaks of the apical four chamber, and the ratio of E/A was calculated.

Blood samples obtained from the abdominal aorta were then centrifuged at 2095 × g for 10 min. The supernatant was collected for determination of cardiac troponin (cTn)‐I levels. After collecting blood samples, rat hearts were dissected and washed with 0.9% cold saline; vessels, and connective tissue around the heart, atrial and right ventricular tissue was separated. Harvested cardiac tissue was cut into two parts: the apical tissues were immediately frozen in liquid nitrogen until Western blot analysis; remaining tissues were fixed in 10% neutral buffered formalin.

### Determination of cardiac enzymes

Cardiac troponin‐I levels were determined by using the AU400 automatic Biochemical Analyzer (Olympus Co., Tokyo, Japan).

### Histological evaluation

Isolated heart issues were paraffin embedded and the tissue specimens were cut into 5 μm sections for subsequent haematoxylin and eosin staining. Masson staining was employed to detect interstitial fibrosis. Images were visualized by using a light microscope (Olympus BX51; Olympus Co.) and digital imaging system (Olympus DP71; Olympus Co.). The haematoxylin and eosin‐stained images were observed at 400× magnification; tissue sections stained with Masson stain were examined at 40× magnifications. The investigators involved in histological evaluation were blinded to the study group. Images of the stained sections were analysed with Image‐Pro Plus 6.0 image analyses software (Media Cybernetics, Rockville, MD, USA). The myocardial collagen volume fraction (CVF) was calculated as the collagen area/total area. We selected five visual fields of per section, took 15 sections from each group and then calculated the mean CVF.

### Detection of apoptotic cells

Apoptotic cells were detected by using a commercially available TUNEL assay kit (Promega Co), as per the manufacturer's instructions. Briefly, sections were deparaffinized with xylene, dehydrated with graded alcohol, digested with proteinase K (20 μg/ml) for 15 min. at room temperature. After immersing in PBS for 5 min., sections were incubated with a terminal deoxynucleotidyl transferase solution in a 37°C humidified room for 60 min. The enzymatic reactions were terminated by soaking sections in the stop buffer and gently washing with PBS. Streptavidin‐horseradish peroxidase solution was added to each section and incubated in a dark chamber for 30 min. at room temperature. After washing with PBS, slides were exposed to 3,3′‐diaminobenzidine (DAB; Golden Bridge Biotechnology, Peking, China) for 5–7 min. After washing, slides were counter stained with haematoxylin. The stained slides were examined under a light microscope (Olympus BX51; Olympus Co.) and analysed with a computer‐assisted colour image analysis system (Image‐ProPlus 7.0; Media Cybernetics). The TUNEL‐positive nuclei of cardiomyocytes were quantified by randomly counting 10 different microscopic fields (×400) for each section. The ratio of apoptotic myocardial cells was calculated by dividing the number of TUNEL‐positive nuclei by the total number of counted nuclei.

### Detection of the presence of GRP78, CHOP and Caspase‐12

Immunohistochemical analysis was performed for assessing the expression of GRP78, CHOP and Caspase‐12. In brief, after deparaffinization, endogenous peroxidases in the sections were blocked with 0.3% hydrogen PBS, followed by treatment with 1% bovine serum albumin for 60 min. at room temperature. Goat monoclonal anti‐GRP78, anti‐CHOP or anti‐Caspase‐12 primary antibodies at 1:50 dilution were added to the sections overnight at 4°C. The sections were washed with PBS and incubated with biotinylated secondary antibody and streptavidin‐horseradish peroxidase solution. After washing with PBS, sections were incubated with DAB (Roche, Mannheim, Germany) for 5 min., and counterstained with haematoxylin. PBS instead of primary antibody was used to serve as the negative control. The stained sections were visualized using a light microscope (Olympus BX51; Olympus Co.) at 400× and analysed with a computer‐assisted colour image analysis system (Image‐ProPlus 6.0; Media Cybernetics). The density of immunopositive nuclei was determined randomly in five microscopic fields per section and the average optical density was calculated for each group.

### Determination of GRP78, CHOP and Caspase‐12 protein levels

Western blot analysis was used to detect GRP78, CHOP and Caspase‐12 protein expression in the myocardial tissues. Briefly, total proteins from cardiomyocytes were extracted as described in a previous study 26 and the protein concentration was determined by the Bradford method. Eighty micrograms of the extracted total protein was separated by 12% SDS‐PAGE and transferred to nitrocellulose membranes. The membranes were blocked with 5% bovine serum albumin for 40 min., followed by incubation with the primary antibodies against GRP78 (1:500 dilution), CHOP (1:500 dilution), Caspase‐12 (1:500 dilution) or β‐actin (1:500 dilution) at 4°C overnight. The membranes were then washed three times with TBS‐Tween and incubated with the appropriate secondary antibody at a dilution of 1:500 for 90 min. Protein contents were detected using an enhanced chemiluminescent reagent. The densitometry of bands was quantified by using Image‐Pro Plus 6.0 system (Media Cybernetics) and expressed relative to the β‐actin bands.

### Data analyses

All results were expressed as mean ± S.D. Comparisons between study groups were made by one‐way analysis of variance, followed by Bonferroni's *post hoc* test. Data were analysed with SPSS (version 17.0) software (SPSS Inc., Chicago, IL, USA), and *P* < 0.05 was considered as statistically significant.

## Results

### Basic characteristics

**Figure 1:**
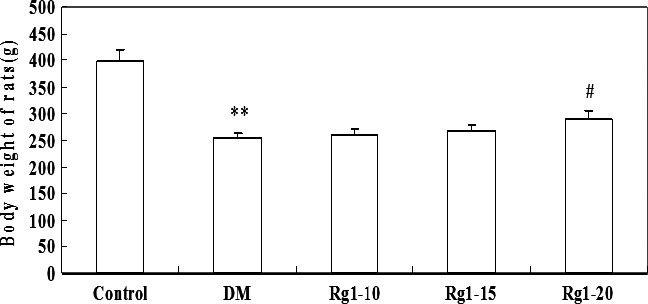
Comparison of bodyweight changes. Data are expressed as mean ± S.D. **P < 0.01 *versus* non‐diabetic control group; *^#^P* < 0.05 *versus* DM group. DM: diabetic model.

Rats in the control group were treated with a normal amount of diet and water intake, and maintained a normal bodyweight and white shiny fur. The rats fed on a diet rich in fat and sugar had a significant increase in bodyweight. After STZ injection, polydipsia, polyuria or polyphagia symptoms gradually worsened with a slower reaction time. Moreover, the fur turned noticeably dry in the diabetic rats. Bodyweight began to decrease after STZ injection; the weight loss was statistically significant as compared to that of the control group at the end of the experiment (*P* < 0.01). Compared with the untreated diabetic rats, rats treated with ginsenoside Rg1 had an increase in bodyweight and the difference in weight was statistically significant in the group treated with high doses of ginsenoside Rg1 (20 mg/kg) (Fig. 1).

### Effects of ginsenoside Rg1 on echocardiographic parameters

Echocardiographic parameters of cardiac function are summarized in Table 1. Compared with the normal control group, LVEF, LVFS and E/A were significantly lower and LVEDD or LVPWD were significantly higher in the untreated diabetic group (all *P* < 0.05). Ginsenoside Rg1 15 or 20 mg/kg treatment were associated with an improvement in LVEF, LVFS, E/A and LVEDD compared with untreated diabetic group (*P* < 0.05 or *P* < 0.01). Ginsenoside Rg1 10 mg/kg treatment was associated with a decreased LVPWD value than those in the untreated diabetic group (*P* < 0.05).

**Table 1:** Comparison of echocardiographic parameters after ginsenoside Rg1 treatment

	LVEDD (mm)	E/A	LVPWD (mm)	LVEF (%)	LVFS (%)
Control	4.34 ± 0.89	2.55 ± 0.38	3.47 ± 0.25	88.21 ± 0.95	52.25 ± 0.84
DM	7.23 ± 0.23^a^	0.32 ± 0.04^a^	5.53 ± 0.51^b^	75.57 ± 5.21^b^	45.52 ± 0.51^b^
Ginsenoside Rg1‐10	5.82 ± 0.39^b^	0.31 ± 0.03^a^	3.31 ± 0.15^c^	74.56 ± 5.69^b^	40.33 ± 8.35^b^
Ginsenoside Rg1‐15	4.57 ± 0.68^c^	1.38 ± 0.09^c^	3.30 ± 0.36^c^	87.36 ± 4.25^c^	52.13 ± 3.56^c^
Ginsenoside Rg1‐20	4.25 ± 0.24^d^	1.25 ± 0.08^c^	2.72 ± 0.29^c^	85.24 ± 1.56^c^	52.58 ± 2.45^c^

a *P* < 0.01 compared with the non‐diabetic control group.

b *P* < 0.05.

c *P* < 0.05.

d *P* < 0.01 compared with the DM group.

Data are expressed as mean ± S.D.

DM: diabetic model; LVEDD: left ventricular end diastolic diameter; LVPWD: left ventricular posterior wall thickness; LVFS: left ventricular shortening fraction; LVEF: left ventricular ejection fraction.

### Effects of ginsenoside Rg1 on serum cTn‐I levels

**Figure 2:**
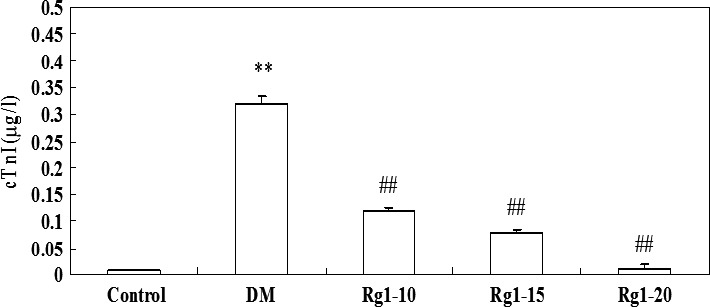
Effects of ginsenoside Rg1 on serum cTn‐I levels. Data are expressed as mean ± S.D. **P < 0.01 *versus* non‐diabetic control group; *^##^P* < 0.01 *versus* DM group. DM: diabetic model; cTn‐I: cardiac troponin‐I.

As shown in Figure 2, serum cTn‐I levels in the untreated diabetic group were significantly higher than those in the normal control group (0.32 ± 0.012 *versus* 0.007 ± 0.003 μg/l, *P* < 0.01). Ginsenoside Rg1 treatment was associated with a decreased cTn‐I levels to 0.12 ± 0.008 μg/l at 10 mg/kg, to 0.08 ± 0.005 μg/l at 15 mg/kg and 0.012 ± 0.006 μg/l at 20 mg/kg, compared with the untreated diabetic group (all *P* < 0.01).

### Effects of ginsenoside Rg1 on myocardial pathological abnormalities

**Figure 3:**
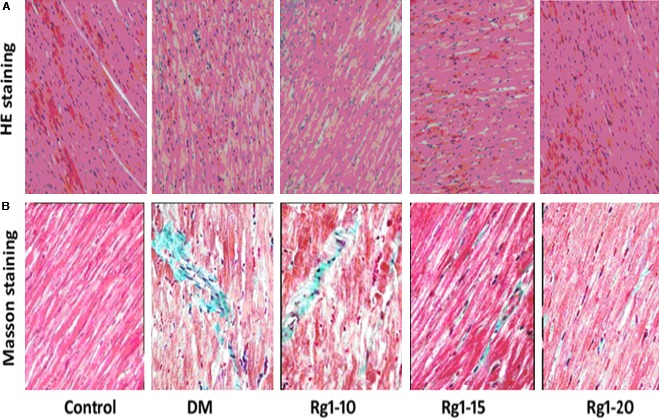
Effects of ginsenoside Rg1 treatment on histopathological abnormalities in myocardial tissue. (**A**) Haematoxylin and eosin staining (400× magnification) showed irregular arrangement of myocardial cells, uneven distribution of cytoplasm and ruptured myocardial fibres in the diabetic model group, while treatment with ginsenoside Rg1 attenuated the above histopathological changes. (**B**) Masson staining (40× magnification) showed irregular and noticeably increased interstitial collagen fibres (green region) in the diabetic model group, while ginsenoside Rg1 20 mg/kg significantly attenuated histopathological changes. DM: diabetic model.

**Figure 4:**
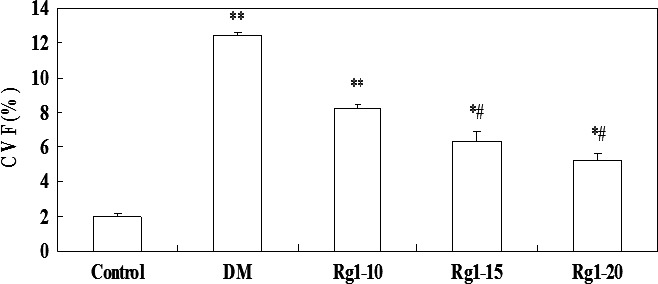
Effects of ginsenoside Rg1 on myocardial collagen volume fraction. Data are expressed as mean ± S.D. *P < 0.05, **P < 0.01 *versus* non‐diabetic control group; *^#^P* < 0.05 *versus* DM group. DM: diabetic model; CVF: collagen volume fraction.

On haematoxylin and eosin staining (Fig. 3A), myocardial cells in the normal control group appeared to be more compact and arranged in an orderly manner, with bright red cytoplasm and centrally located oval nuclei. No dissolved muscle fibres or any evidence of vacuolar degeneration and mononuclear cell infiltration were observable. However, in the untreated diabetic group, disorderly arranged myocardial cells, uneven cytoplasm distribution, rupture of myocardial fibres and irregular nuclei were visualized. Ginsenoside Rg1 treatment attenuated the above myocardial injury, particularly in rats that were administered high doses of ginsenoside Rg1. As shown on Masson staining (Fig. 3B), in the normal control group, intact collagen fibres and no obvious myocardial interstitial collagen deposition were found. In the untreated diabetic group, myocardial cells showed an irregular arrangement and increased interstitial collagen fibres in the intercellular and perivascular space. Ginsenoside Rg1 treatment reduced the collagen fibres (green belt), particularly in high‐dose ginsenoside Rg1 rats. As shown in Figure 4, CVF in the untreated diabetic group significantly increased compared with the control group (*P* < 0.01). Ginsenoside Rg1 15 or 20 mg/kg treatment was associated with a decreased CVF (all *P* < 0.05).

### Effect of ginsenoside Rg1 on myocardial cell apoptosis

**Figure 5:**
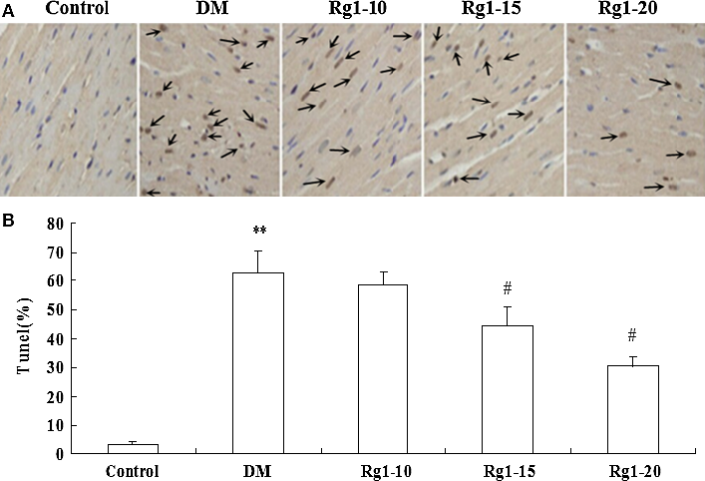
Effects of ginsenoside Rg1 treatment on cardiomyocytes apoptosis. (**A**) Images of TUNEL staining in different groups. Apoptotic nuclei were stained dark brown (denoted by arrows; scale bar: 15 µm); (**B**) apoptotic cells expressed as a percentage of TUNEL‐positive nuclei number/total number of counted nuclei ×100%. Data are expressed as mean ± S.D. (*N* = 15). **P < 0.01 *versus* non‐diabetic control group; *^#^P* < 0.05 *versus* DM group. DM: diabetic model.

As shown in Figure 5A, there were no TUNEL‐positive myocardial cells in the control group; evidence of increased myocardial cell apoptosis was observed in untreated diabetic rats. The percentage of apoptotic myocardial cells in the untreated diabetic group was significantly higher than that in the normal control group (62.5 ± 7.59% *versus* 3.23 ± 1.32%, *P* < 0.01) (Fig. 5B). Ginsenoside Rg1 treatment was associated with a decreased percentage of apoptotic myocardial cells to 58.23 ± 4.65% at 10 mg/kg, to 44.25 ± 6.58% at 15 mg/kg and 30.68 ± 2.88% at 20 mg/kg, compared with the untreated diabetic group (*P* < 0.05 at 15 or 20 mg/kg).

### GRP78, CHOP and Caspase‐12 expression by immunohistochemical analysis

**Figure 6:**
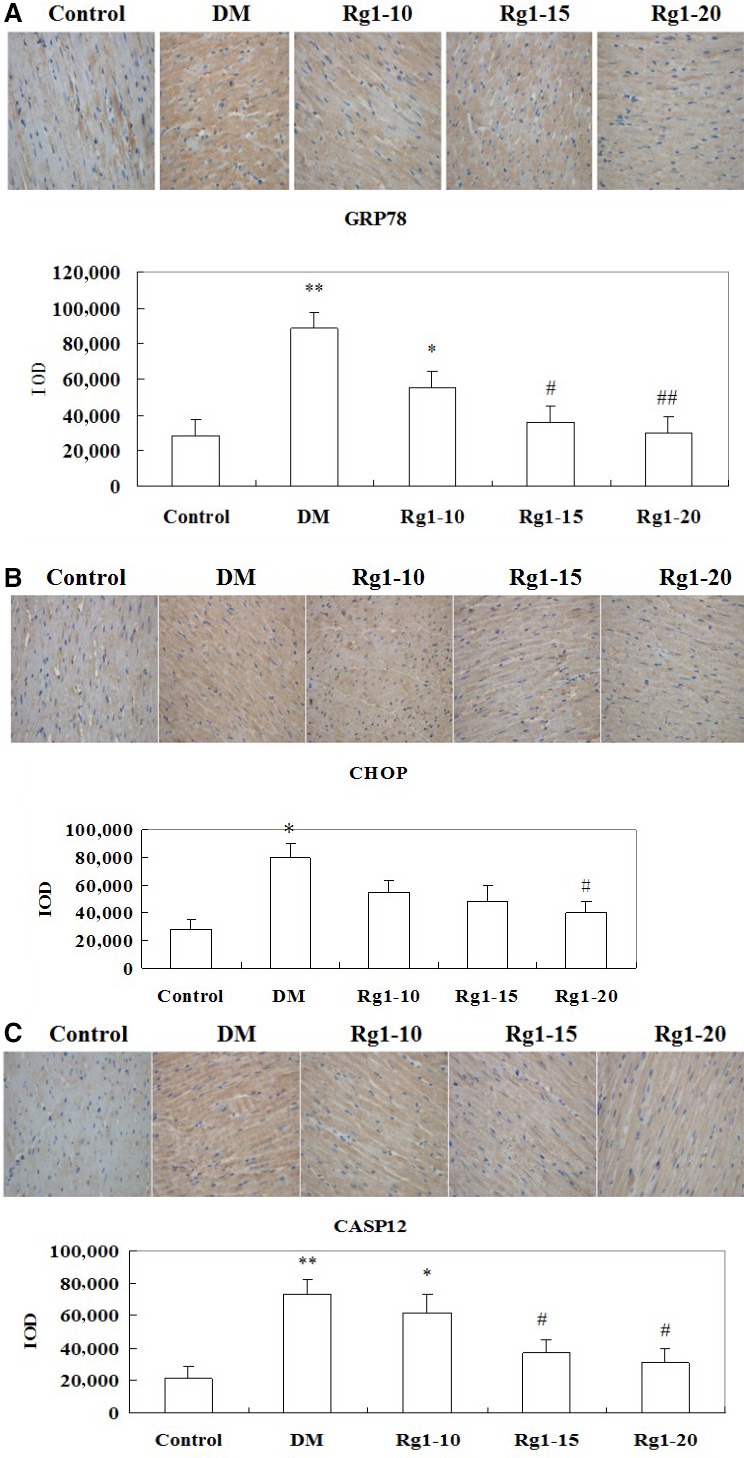
Effects of ginsenoside Rg1 treatment on GRP78 (**A**), CHOP (**B**) and Caspase‐12 (**C**) expression. Upper images represent the immunohistochemical staining in different groups (400× magnification). The positive immunohistochemical stainings are presented as yellowish brown. Lower graphs represent the results of optical density analysis. Data are expressed as mean ± S.D. (*N* = 15). *P < 0.05, **P < 0.01 *versus* non‐diabetic control group; *^#^P* < 0.05, *^##^P* < 0.01 *versus* DM group. DM: diabetic model; CASP: Caspase.

In Figure 6A, the yellow or brown staining of the cytoplasm represents positive immunostaining for GRP78. On optical density analysis, positive staining optical density of GRP78 was significantly higher in the untreated diabetic group than in the normal control group (*P* < 0.01). Treatment with ginsenoside Rg1 reduced the positive staining optical density of GRP78 in a dose‐dependent manner. The positive staining of CHOP was manifested as yellow or brown in the cytoplasm (Fig. 6B). On optical density analysis, positive staining optical density of CHOP was significantly higher in the untreated diabetic group than in the normal control group (*P* < 0.01). Ginsenoside Rg1 treatment resulted in a decreased positive staining optical density of CHOP in a dose‐dependent manner (Fig. 6C). The positive staining of Caspase‐12 manifested itself in yellow or brown colour in the cytoplasm. Optical density analysis showed that Caspase‐12 expression was significantly higher in the untreated diabetic group than in the normal control group (*P* < 0.01). The findings suggest that ginsenoside Rg1 treatment was associated with a reduction in Caspase‐12 expression in a dose‐dependent manner.

### GRP78, CHOP and Caspase‐12 protein levels by Western blot analysis

**Figure 7:**
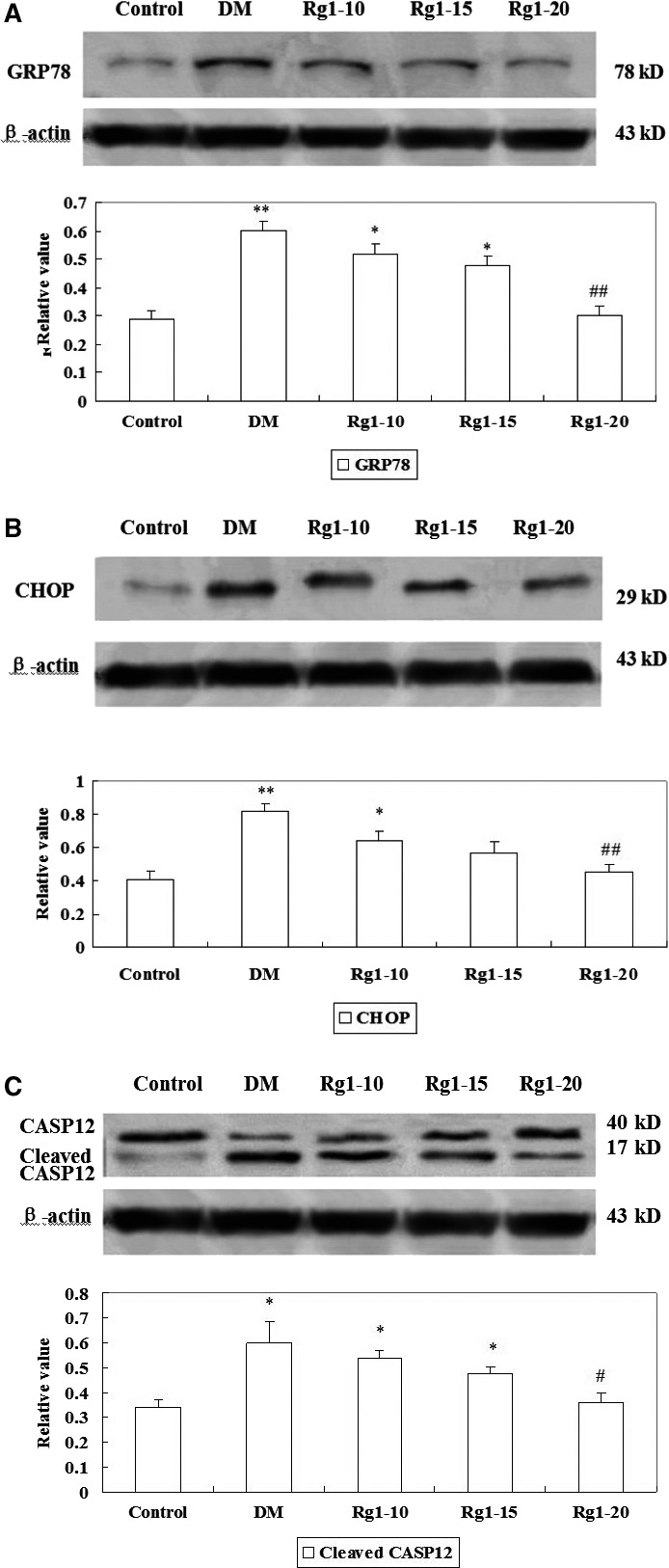
Effects of ginsenoside Rg1 treatment on GRP78 (**A**), CHOP (**B**) and Caspase‐12 (**C**) protein levels. Western blot was conducted using total protein from cardiomyocytes. Upper images represent protein band in different groups. Lower graphs represent the bar graphs summarizing the immunoblot data. Western blot was performed using each relevant antibody. β‐Actin was shown as a loading control. Data are expressed as mean ± S.D. (*N* = 15). *P < 0.05, **P < 0.01 *versus* non‐diabetic control group; *^#^P* < 0.05, *^##^P* < 0.01 *versus* DM group. DM: diabetic model; CASP: Caspase.

Representative GRP78 band with a molecular weight of 78 kD of Western blot analysis is shown in Figure 7A. Myocardial GRP78 protein level was increased in untreated diabetic rats compared with normal control rats (*P* < 0.01). Ginsenoside Rg1 20 mg/kg treatment significantly decreased myocardial GRP78 protein level than in the diabetic rat models (*P* < 0.01). Figure 7B shows the representative CHOP band with a molecular weight of 29 kD on Western blot analysis. An increased CHOP protein level was observed in untreated diabetic rats as compared to the normal controls (*P* < 0.01). Ginsenoside Rg1 20 mg/kg treatment significantly decreased CHOP protein level in the myocardial tissues as compared to that in the untreated diabetic rats (*P* < 0.01). Further, the normal rat myocardium showed Caspase‐12 band with a molecular weight of 40 kD, and a weak band with a molecular weight of 17 kD. An increase in cleaved Caspase‐12 protein level was observed in untreated diabetic rats as compared to that in the normal controls (*P* < 0.05). Ginsenoside Rg1 20 mg/kg treatment significantly reduced cleaved Caspase‐12 protein level in the myocardial tissues than those in the untreated diabetic rats (*P* < 0.05; Fig. 7C).

## Discussion

In the current study, we established a diabetes model with a diet rich in fat and sugar content, and STZ 40 mg/kg injection. Histological examination of the haematoxylin and eosin and Masson‐stained sections of diabetic rat heart tissue showed disordered myocardial cells, rupture of myocardial fibres and interstitial fibrosis. Diabetic cardiac hypertrophy was demonstrated by echocardiography. Occurrence of myocardial insult was substantiated by elevated cardiac enzyme levels. TUNEL staining demonstrated enhanced apoptosis in cardiocytes in diabetic rats. The key findings emanating from this study are that ginsenoside Rg1 treatment was associated with attenuation of histopathological changes in diabetic myocardium; reduced serum cTnI levels and the biochemical markers of myocardial cellular injury; improved parameters of cardiac function; decreased myocardial cell apoptosis; reduced GRP78, CHOP and cleavage of Caspase‐12 protein expression. Our previous study 27 indicated that there were no statistically significant changes in the serum levels of fasting blood glucose (FBG), total cholesterol (TC) and triglyceride (TG). The findings suggest that ginsenoside Rg1 attenuated diabetic myocardial damage in STZ‐induced diabetic rats through reducing the CHOP/Caspase‐12‐induced apoptosis in ER stress.

The underlying mechanism of diabetic cardiomyopathy is complex 28. Hyperglycaemia‐induced apoptosis in cardiomyocytes is considered to be an important mechanism of diabetic cardiomyopathy 29. Multiple pathological stimuli including ischaemia, oxidative stress, hypoxia, hyperglycaemia, and hyperlipidaemia are known to cause ER stress. However, exposure to high glucose levels appears to be the central mechanism of ER stress 30. Endoplasmic reticulum plays a crucial role in cellular homeostasis. Excessive or prolonged ER stress can eventually trigger cell apoptosis 31. Activation of GRP78 protein expression is a well‐established marker of ER stress. Endoplasmic reticulum stress‐related apoptotic signalling proteins include CHOP, Caspase‐12 and c‐JunN‐terminalkinase (JNK) pathway. Of these three signalling proteins, Caspase‐12 and CHOP are specific apoptotic pathways of ER. In this study, upregulation of GRP78, CHOP and cleaved Caspase‐12 protein expression was found in the diabetic myocardium, which indicates that excessive ER stress was involved in the pathology of diabetic cardiomyopathy. Treatment with ginsenoside Rg1 attenuated diabetic myocardial injury as well as decreased apoptosis in cardiocytes. In addition, ginsenoside Rg1 treatment appeared to reduce GRP78 and CHOP, and cleaved Caspase‐12 protein expression in a dose‐dependent manner. Together these findings suggest that the cardioprotective effect of ginsenoside Rg1 may be mediated through the ER stress‐induced apoptosis. Several studies have investigated the cardioprotective effects of ginsenoside Rg1. Ginsenoside Rg1 has been shown to prevent rat LV hypertrophy and cardiac dysfunction 23, protect rat cardiomyocytes from hypoxia/reoxygenation oxidative injury 32, prolong ventricular refractoriness and repolarization in dogs 33, attenuate rat myocardial injury induced by ischaemia and reperfusion 25, and protect high glucose‐induced myocardial hypertrophy 34. Various properties including antioxidation, anti‐apoptosis and inhibiting ER stress have been attributed to the cardioprotective effects of ginsenoside Rg1 22. In addition to inhibiting ER stress‐induced apoptosis in diabetic rats, ginsenoside Rg1 treatment also reduced neuron 35, tubular cell 36, lymphocyte 37, PC12 cell 38, human endothelial cell 39 and Jurkat cell apoptosis 40. Therefore, downregulation of excessive cell apoptosis appears to play a dominant role in the cardioprotective mechanism.

Some potential limitations of our study need to be taken into account while interpreting the study results. First, STZ‐induced DM is a reflection of type 1 diabetes rather than type 2 diabetes 41. Therefore, the current diabetic rat model might not be representative of the pathologic processes in type 2 diabetes. More preclinical studies based on type 2 diabetic rat models are needed to substantiate these results. Secondly, ER is known to interplay with mitochondria in regulating apoptosis; however, we did not address the role of the mitochondrial pathway in this study. Further studies are needed to elucidate any additional signal pathways including mitochondrial pathway that may be involved in mediating the cardioprotective effects of ginsenoside Rg1 treatment.

In summary, the present study indicates that ER stress‐induced apoptosis plays a role in the pathophysiology of diabetic cardiomyopathy. Ginsenoside Rg1 treatment attenuated diabetic myocardial damage in STZ‐induced diabetic rats by reducing ER stress‐induced apoptosis. Our findings reveal a novel mechanism of action of ginsenoside Rg1 in the management of diabetic cardiomyopathy.

## Conflicts of interest

Haitao Yu, Juan Zhen, Yang Yang, Jinning Gu, Suisheng Wu and Quan Liu declare that they have no conflicts of interest.

## References

[1] Boudina S , Abel ED . Diabetic cardiomyopathy revisited. Circulation. 2007; 115: 3213–23. 17592090 10.1161/CIRCULATIONAHA.106.679597

[2] Go AS , Mozaffarian D , Roger VL , *et al* Heart disease and stroke statistics–2014 update: a report from the American Heart Association. Circulation. 2014; 129: e28–292. 24352519 10.1161/01.cir.0000441139.02102.80 PMC5408159

[3] Dandamudi S , Slusser J , Mahoney DW , *et al* The prevalence of diabetic cardiomyopathy: a population‐based study in Olmsted County, Minnesota. J Card Fail. 2014; 20: 304–9. 24576788 10.1016/j.cardfail.2014.02.007 PMC4076144

[4] Miki T , Yuda S , Kouzu H , *et al* Diabetic cardiomyopathy: pathophysiology and clinical features. Heart Fail Rev. 2013; 18: 149–66. 22453289 10.1007/s10741-012-9313-3 PMC3593009

[5] Hostiuc S , Popescu A , Gutu ED , *et al* Electrical conduction system apoptosis in type II diabetes mellitus. Rom J Morphol Embryol. 2013; 54: 953–9. 24398990

[6] Cai L , Kang YJ . Cell death and diabetic cardiomyopathy. Cardiovasc Toxicol. 2003; 3: 219–28. 14555788 10.1385/ct:3:3:219

[7] Kumar S , Prasad S , Sitasawad SL . Multiple antioxidants improve cardiac complications and inhibit cardiac cell death in streptozotocin‐induced diabetic rats. PLoS ONE. 2013; 8: e67009. 23843977 10.1371/journal.pone.0067009 PMC3699585

[8] Jackson CV , McGrath GM , Tahiliani AG , *et al* A functional and ultrastructural analysis of experimental diabetic rat myocardium. Manifestation of a cardiomyopathy. Diabetes. 1985; 34: 876–83. 3896897 10.2337/diab.34.9.876

[9] Bhimji S , Godin DV , McNeill JH . Myocardial ultrastructural changes in alloxan‐induced diabetes in rabbits. Acta Anat. 1986; 125: 195–200. 3962581 10.1159/000146161

[10] Xu J , Zhou Q , Xu W , *et al* Endoplasmic reticulum stress and diabetic cardiomyopathy. Exp Diabetes Res. 2012; 2012: 827971. 22144992 10.1155/2012/827971 PMC3226330

[11] Li Z , Zhang T , Dai H , *et al* Involvement of endoplasmic reticulum stress in myocardial apoptosis of streptozocin‐induced diabetic rats. J Clin Biochem Nutr. 2007; 41: 58–67. 18392099 10.3164/jcbn.2007008 PMC2274987

[12] Szegezdi E , Logue SE , Gorman AM , *et al* Mediators of endoplasmic reticulum stress‐induced apoptosis. EMBO Rep. 2006; 7: 880–5. 16953201 10.1038/sj.embor.7400779 PMC1559676

[13] Zhao Y , Yan Y , Zhao Z , *et al* The dynamic changes of endoplasmic reticulum stress pathway markers GRP78 and CHOP in the hippocampus of diabetic mice. Brain Res Bull. 2015; 111: 27–35. 25529350 10.1016/j.brainresbull.2014.12.006

[14] Li J , Holbrook NJ . Elevated gadd153/chop expression and enhanced c‐Jun N‐terminal protein kinase activation sensitizes aged cells to ER stress. Exp Gerontol. 2004; 39: 735–44. 15130668 10.1016/j.exger.2004.02.008

[15] Wang XZ , Lawson B , Brewer JW , *et al* Signals from the stressed endoplasmic reticulum induce C/EBP‐homologous protein (CHOP/GADD153). Mol Cell Biol. 1996; 16: 4273–80. 8754828 10.1128/mcb.16.8.4273 PMC231426

[16] Hetz C , Russelakis‐Carneiro M , Maundrell K , *et al* Caspase‐12 and endoplasmic reticulum stress mediate neurotoxicity of pathological prion protein. EMBO J. 2003; 22: 5435–45. 14532116 10.1093/emboj/cdg537 PMC213791

[17] Younce CW , Burmeister MA , Ayala JE . Exendin‐4 attenuates high glucose‐induced cardiomyocyte apoptosis *via* inhibition of endoplasmic reticulum stress and activation of SERCA2a. Am J Physiol Cell Physiol. 2013; 304: C508–18. 23302777 10.1152/ajpcell.00248.2012

[18] Xu J , Wang G , Wang Y , *et al* Diabetes‐ and angiotensin II‐induced cardiac endoplasmic reticulum stress and cell death: metallothionein protection. J Cell Mol Med. 2009; 13: 1499–512. 19583814 10.1111/j.1582-4934.2009.00833.x PMC3828862

[19] Liu X , Xu Q , Wang X , *et al* Irbesartan ameliorates diabetic cardiomyopathy by regulating protein kinase D and ER stress activation in a type 2 diabetes rat model. Pharmacol Res. 2015; 93: 43–51. 25617729 10.1016/j.phrs.2015.01.001

[20] Cicek FA , Toy A , Tuncay E , *et al* Beta‐blocker timolol alleviates hyperglycemia‐induced cardiac damage *via* inhibition of endoplasmic reticulum stress. J Bioenerg Biomembr. 2014; 46: 377–87. 25064604 10.1007/s10863-014-9568-6

[21] Nah JJ , Hahn JH , Chung S , *et al* Effect of ginsenosides, active components of ginseng, on capsaicin‐induced pain‐related behavior. Neuropharmacology. 2000; 39: 2180–4. 10963761 10.1016/s0028-3908(00)00048-4

[22] Lee CH , Kim JH . A review on the medicinal potentials of ginseng and ginsenosides on cardiovascular diseases. J Ginseng Res. 2014; 38: 161–6. 25378989 10.1016/j.jgr.2014.03.001 PMC4213864

[23] Zhang YJ , Zhang XL , Li MH , *et al* The ginsenoside Rg1 prevents transverse aortic constriction‐induced left ventricular hypertrophy and cardiac dysfunction by inhibiting fibrosis and enhancing angiogenesis. J Cardiovasc Pharmacol. 2013; 62: 50–7. 23846802 10.1097/FJC.0b013e31828f8d45

[24] Wang XD , Gu TX , Shi EY , *et al* Effect and mechanism of panaxoside Rg1 on neovascularization in myocardial infarction rats. Chin J Integr Med. 2010; 16: 162–6. 20473743 10.1007/s11655-010-0162-4

[25] Li X , Chen JX , Sun JJ . Protective effects of Panax notoginseng saponins on experimental myocardial injury induced by ischemia and reperfusion in rat. Zhongguo Yao Li Xue Bao. 1990; 11: 26–9. 2403009

[26] Xiuhua L , Xudong W , Yue H , *et al* Signaling pathway of cardioprotection induced by monophosphoryl lipid A in rabbit myocardium. Pathophysiology. 2002; 8: 193–6. 12039651 10.1016/s0928-4680(02)00005-6

[27] Yu HT , Zhen J , Pang B , *et al* Ginsenoside Rg1 ameliorates oxidative stress and myocardial apoptosis in streptozotocin‐induced diabetic rats. J Zhejiang Univ Sci B. 2015; 16: 344–54. 25990051 10.1631/jzus.B1400204 PMC4432986

[28] Bugger H , Abel ED . Molecular mechanisms of diabetic cardiomyopathy. Diabetologia. 2014; 57: 660–71. 24477973 10.1007/s00125-014-3171-6 PMC3969857

[29] Huynh K , Bernardo BC , McMullen JR , *et al* Diabetic cardiomyopathy: mechanisms and new treatment strategies targeting antioxidant signaling pathways. Pharmacol Ther. 2014; 142: 375–415. 24462787 10.1016/j.pharmthera.2014.01.003

[30] Lakshmanan AP , Harima M , Suzuki K , *et al* The hyperglycemia stimulated myocardial endoplasmic reticulum (ER) stress contributes to diabetic cardiomyopathy in the transgenic non‐obese type 2 diabetic rats: a differential role of unfolded protein response (UPR) signaling proteins. Int J Biochem Cell Biol. 2013; 45: 438–47. 23032698 10.1016/j.biocel.2012.09.017

[31] Rao RV , Ellerby HM , Bredesen DE . Coupling endoplasmic reticulum stress to the cell death program. Cell Death Differ. 2004; 11: 372–80. 14765132 10.1038/sj.cdd.4401378

[32] Zhu D , Wu L , Li CR , *et al* Ginsenoside Rg1 protects rat cardiomyocyte from hypoxia/reoxygenation oxidative injury *via* antioxidant and intracellular calcium homeostasis. J Cell Biochem. 2009; 108: 117–24. 19530220 10.1002/jcb.22233

[33] Wu W , Zhang XM , Liu PM , *et al* Effects of Panax notoginseng saponin Rg1 on cardiac electrophysiological properties and ventricular fibrillation threshold in dogs. Zhongguo Yao Li Xue Bao. 1995; 16: 459–63. 8701769

[34] Li TC , Wang HX . Effects of ginsenoside Rg1 on high glucose‐induced myocardial hypertrophy. Pharmacol Clin Chin Mater Med. 2014; 30: 25–7.

[35] Wang B , He L , Cui B , *et al* Protection of ginsenoside Rg1 on central nerve cell damage and the influence on neuron apoptosis. Pak J Pharm Sci. 2014; 27: 2035–40. 25410069

[36] Liu QF , Deng ZY , Ye JM , *et al* Ginsenoside rg1 protects chronic cyclosporin a nephropathy from tubular cell apoptosis by inhibiting endoplasmic reticulum stress in rats. Transplant Proc. 2015; 47: 566–9. 25769608 10.1016/j.transproceed.2014.10.047

[37] Zou Y , Tao T , Tian Y , *et al* Ginsenoside Rg1 improves survival in a murine model of polymicrobial sepsis by suppressing the inflammatory response and apoptosis of lymphocytes. J Surg Res. 2013; 183: 760–6. 23478085 10.1016/j.jss.2013.01.068

[38] Huang SL , He XJ , Li ZF , *et al* Neuroprotective effects of ginsenoside Rg1 on oxygen‐glucose deprivation reperfusion in PC12 cells. Pharmazie. 2014; 69: 208–11. 24716411

[39] Yan J , Liu Q , Dou Y , *et al* Activating glucocorticoid receptor‐ERK signaling pathway contributes to ginsenoside Rg1 protection against beta‐amyloid peptide‐induced human endothelial cells apoptosis. J Ethnopharmacol. 2013; 147: 456–66. 23538162 10.1016/j.jep.2013.03.039

[40] Li H , Xu J , Wang X , *et al* Protective effect of ginsenoside Rg1 on lidocaine‐induced apoptosis. Mol Med Rep. 2014; 9: 395–400. 24270314 10.3892/mmr.2013.1822 PMC3896509

[41] Gur S , Peak TC , Kadowitz PJ , *et al* Review of erectile dysfunction in diabetic animal models. Curr Diabetes Rev. 2014; 10: 61–73. 24295372 10.2174/1573399809666131126151024

